# Management of refractory hypoxemia using recruitment maneuvers and rescue therapies: A comprehensive review

**DOI:** 10.3389/fvets.2023.1157026

**Published:** 2023-03-30

**Authors:** Félix Bajon, Vincent Gauthier

**Affiliations:** Centre Vétérinaire Laval, Laval, QC, Canada

**Keywords:** acute respiratory distress syndrome (ARDS), recruitment maneuvers, refractory hypoxemia, rescue therapies, ventilator-induced lung injury, airway pressure release ventilation (APRV), veterinary

## Abstract

Refractory hypoxemia in patients with acute respiratory distress syndrome treated with mechanical ventilation is one of the most challenging conditions in human and veterinary intensive care units. When a conventional lung protective approach fails to restore adequate oxygenation to the patient, the use of recruitment maneuvers and positive end-expiratory pressure to maximize alveolar recruitment, improve gas exchange and respiratory mechanics, while reducing the risk of ventilator-induced lung injury has been suggested in people as the open lung approach. Although the proposed physiological rationale of opening and keeping open previously collapsed or obstructed airways is sound, the technique for doing so, as well as the potential benefits regarding patient outcome are highly controversial in light of recent randomized controlled trials. Moreover, a variety of alternative therapies that provide even less robust evidence have been investigated, including prone positioning, neuromuscular blockade, inhaled pulmonary vasodilators, extracorporeal membrane oxygenation, and unconventional ventilatory modes such as airway pressure release ventilation. With the exception of prone positioning, these modalities are limited by their own balance of risks and benefits, which can be significantly influenced by the practitioner's experience. This review explores the rationale, evidence, advantages and disadvantages of each of these therapies as well as available methods to identify suitable candidates for recruitment maneuvers, with a summary on their application in veterinary medicine. Undoubtedly, the heterogeneous and evolving nature of acute respiratory distress syndrome and individual lung phenotypes call for a personalized approach using new non-invasive bedside assessment tools, such as electrical impedance tomography, lung ultrasound, and the recruitment-to-inflation ratio to assess lung recruitability. Data available in human medicine provide valuable insights that could, and should, be used to improve the management of veterinary patients with severe respiratory failure with respect to their intrinsic anatomy and physiology.

## 1. Introduction

Refractory hypoxemia refers to the presence of inadequate oxygenation despite normal levels of inspired oxygen, but no single definition currently achieves consensus amongst the scientific community, either in human or veterinary medicine (1). It is commonly described in humans as either a partial pressure of oxygen (PaO_2_) <60 mm Hg or a PaO_2_/FiO_2_ ratio below 100 mm Hg on a fraction of inspired oxygen (FiO_2_) of 0.8–1.0 with a positive end-expiratory pressure (PEEP) of at least 10–30 cmH_2_O and the inability to maintain a plateau pressure below 30 cmH_2_O (2, 3). Some common causes of refractory hypoxemia include sepsis, pneumonia, major trauma, drowning, burns, and most frequently, acute respiratory distress syndrome (ARDS) (4). Although the main cause of death in ARDS is multiple organ dysfunction syndrome, it is estimated that 10–15% of affected human patients die from refractory hypoxemia (5).

As per the Berlin definition, ARDS is defined in humans as an acute onset of respiratory difficulty and severe hypoxemia (PaO_2_/FiO_2_ ratio <300 mm Hg with a PEEP level ≥ 5 cmH_2_O) occurring within 1 week of a known clinical insult or new or worsening respiratory symptoms, with bilateral opacities consistent with pulmonary edema not fully explained by heart failure or volume overload on chest radiographs or computed tomography (6). In contrast, the current VetALI and VetARDS definitions, designed for veterinary patients, do not incorporate PEEP for the diagnosis of ARDS (7). Pathophysiologically, ARDS is characterized by altered alveolar permeability, accumulation of pulmonary edema and decreased functional residual capacity, causing decreased respiratory compliance and hypoxemia (8). Respiratory support by mechanical ventilation is essential in the management of ARDS to maintain an adequate level of oxygenation and ventilation (9). One of the main challenges with managing these patients lies in the fact that mechanical ventilation, although aimed precisely at recruiting these non-aerated lung regions, can contribute to the worsening of pulmonary lesions, a phenomenon referred to as ventilator-induced lung injury (VILI) (10). The development of VILI then becomes an iatrogenic cause of mortality in patients with ARDS (10).

Ventilation strategies have thus evolved from the optimization of gas exchange to lung protective strategies aimed at limiting atelectrauma, shear-stress, and barotrauma, all three related to the heterogeneous properties of the different lung regions (dependent, transitional, and nondependent, respectively), but also to prevent adverse hemodynamic consequences of positive pressure ventilation (11, 12). Targets that could aggravate or attenuate VILI, in particular tidal volume and PEEP, have become major areas of interest (13).

The open-lung concept is a variant of lung protective ventilation, which combines the use of alveolar recruitment maneuvers to open-up unventilated alveoli and the subsequent use of higher PEEP to maintain alveolar stability and minimize shear-stress (14, 15). From a physiological standpoint, this concept may seem appealing, but the clinical evidence in favor of its use is inconsistent (16–19). In the landmark ARMA trial, a strategy with low tidal volume and limited plateau pressure (<35 cmH_2_O) was linked to reduced mortality in humans with ARDS (11). Similarly, in a different study, a higher PEEP was associated with decreased mortality, but only if this resulted in a decrease in driving pressure (20). Conversely, the recent ART trial concluded that overdistension of alveoli caused by recruitment maneuvers and a higher PEEP could be more harmful than beneficial and be the cause of increased mortality in human patients with moderate to severe ARDS (21). As a result of this important study, several editorials have since suggested strong reconsideration of the open lung approach (22, 23).

Among the various strategies to minimize the size of unavailable lung regions, avoid atelectasis in both diseased and healthy lung settings, and prevent lung overdistension, recruitment maneuvers remain one of the most controversial of all strategies and have been widely studied (18, 19, 24–26). No concrete guidelines on the ideal method to use and the optimal pathological setting in which recruitment maneuvers might demonstrate utility are established due to the lack of high-quality randomized controlled trials (15, 18). The controversy over the efficacy of recruitment maneuvers in the context of refractory hypoxemia also rests on the actual definition of responders and non-responders to these maneuvers, due to the multiplicity of assessment criteria (gas exchange, lung mechanics, hemodynamics) and quantification methods (27–29). Until recently, clinicians lacked a reliable and easy-to-use bedside tool to not only reliably predict lung recruitability, but also to quantify the effectiveness of recruitment maneuvers (27).

The objective of this review is to propose a framework for the rational and safe use of recruitment maneuvers in the context of refractory hypoxemia in patients with ARDS treated with mechanical ventilation. After briefly reviewing the pathophysiology of ARDS and the derecruitment that occurs in acutely injured lungs, the main recruitment methods available will be described, including their advantages and disadvantages, along with ways to quantify the response to recruitment maneuvers. Finally, a focus on alternative mechanical ventilation modes and rescue therapies that can be implemented with refractory hypoxemia will be provided, with a summary on the application of these methods in veterinary medicine.

## 2. Recruitment and derecruitment in acute respiratory distress syndrome

The lungs are inherently a physiologically heterogeneous environment (30). Regional inequalities in ventilation and perfusion related to different factors (gravity, posture, obesity) exist physiologically within the lungs, and constitute a challenge, especially in patients with ARDS requiring ventilatory support (8).

In a spontaneously breathing patient, more pronounced diaphragmatic contractions in the dorsal region as well as sigh breaths optimize alveolar recruitment, maintain pulmonary compliance and, consequently, decrease formation of atelectasis (8). Induction of general anesthesia abolishes these physiological mechanisms, which partly explains why all patients under general anesthesia have various degrees of pulmonary atelectasis (31). This appears to be one of the main mechanisms of acute lung injury, is a major cause of postoperative hypoxemia, and is associated with a prolonged intensive care unit and hospital stay in people (32). Intraoperative effects of atelectasis include increased alveolar-arterial oxygen gradient, increased pulmonary shunting, and decreased oxygen saturation (31). This is a continuous process, independent of previous lung volumes, especially below a PEEP of 15 cmH_2_O (8).

During positive pressure ventilation, three distinct lung regions can be distinguished because the pressure required to open the alveoli, called the threshold opening pressure, varies along the gravity axis (8). In the lower portion of the lungs, the weight of mediastinal structures increases pleural pressure (making it less negative) and thus reduces alveolar volume (8). Smaller alveolar volumes mean that the alveoli have more potential for distension, but are less compliant, and therefore able to exchange less oxygen (8). This area is called the dependent portion of the lungs. Conversely, the upper (non-dependent) part of the lungs includes regions that remain inflated throughout tidal ventilation and can be overinflated by tidal volumes >6 mL/kg and plateau pressures exceeding 30–32 cmH_2_O (8). Although this region has a higher partial pressure of oxygen and ventilates more efficiently due to higher compliance, smaller volumes are exchanged in case of overdistension (8).

In the context of respiratory failure and with the desire to maintain adequate oxygenation, understanding that structural heterogeneity is an intrinsic property of the lung and recognizing the factors that can exacerbate this phenomenon are essential (10). An acutely injured lung comprises a heterogeneous environment of non-aerated areas (consolidated or collapsed), poorly aerated zones and overdistended regions, each with varying recruitability (8). Any positive pressure insufflation creates a risk of overdistension of healthy alveoli and shear stresses at the junction between the ventilated and non-ventilated areas, and causes inflammation and epithelial damage in the small airways and alveoli repeatedly mobilized (9, 11). The latter phenomenon, also known as atelectrauma, together with overdistension, make up the two main independent components of VILI (10). All these phenomena participate in increased cytokine release and contribute to the risk of multiorgan failure and mortality (10). Furthermore, surfactant impairment and increased lung weight in severe ARDS significantly increase alveolar opening pressure due to increased tensile forces (13).

## 3. Definition and rationale for the use of recruitment maneuvers

Mechanical ventilation settings aimed at minimizing VILI are referred to as “protective mechanical ventilation” (11). Lung protective ventilation is the central focus of ventilatory management of patients with ARDS (9). This approach recommends the use of low tidal volumes (4–8 mL/kg of predicted body weight) and low plateau pressures (up to 30–32 cmH_2_O) to reduce alveolar stretch injury associated with repetitive opening and closing of atelectatic alveoli and tidal overdistention (11). It is associated with a significant reduction in mortality and duration of mechanical ventilation in people (11). Failure to implement a lung protective strategy in mechanically ventilated patients with ARDS could significantly increase the risk of VILI (elevated transpulmonary pressure), leading to barotrauma, volutrauma, alveolar rupture and the development of pulmonary edema (8, 33). However, this approach is also associated with increased alveolar derecruitment despite moderate levels of PEEP (8, 34). A lung recruitment maneuver is a dynamic and transient increase in transpulmonary pressure that aims to reverse lung collapse (which consequently does not participate in gas exchange), improve lung compliance, increase end-expiratory lung volume, and improve gas exchange (8–10). The open lung concept is a ventilatory strategy complementary to the protective ventilation notion that aims to reduce atelectrauma and shear stress by performing recruitment maneuvers and subsequently using higher PEEP, thereby maintaining the lung open (12–14, 16). Ultimately, improved gas exchange should produce a decrease in the required FiO_2_ and minimize the risk of oxygen toxicity and organ dysfunction due to hypoxia and hypercapnia (10).

Experimental and clinical studies in animals have shown improved lung mechanics and gas exchange following recruitment maneuvers (35). However, it is important to remember that anatomic recruitment, defined as the restoration of aeration as assessed by computed tomography, often does not coincide with functional recruitment, generally defined as the improvement of gas exchange (36). Moreover, in some cases, increasing the inspiratory pressure could worsen the intrapulmonary shunting by increasing perfusion of collapsed lung areas without improving ventilation (9). The recruitment maneuver may prove ineffective when the applied pressure is insufficient or because the sufficient pressure is excessive. Therefore, anatomic and functional lung recruitment can only coincide if restoration of ventilation of the lung units occurs without alteration of perfusion of those same units (36).

The value of recruitment maneuvers has been extensively studied over the past 30 years. In the case of ARDS in particular, the interpretation of studies of recruitment maneuvers is hampered by several confounding factors: these include the heterogeneous nature of the lung injury among patients, the timing of recruitment maneuvers relative to the onset of the syndrome, the ventilatory strategy chosen and the hemodynamic status of the patient relative to the use of vasoactive drugs that may affect the pulmonary blood flow distribution (8). Thus, important unknowns remain regarding the clinical contexts in which recruitment maneuvers are indicated, how to best perform them, and the very definition of a responder to said maneuver, for which the criteria differ among authors (28).

## 4. Assessing lung recruitability

It is established that the effectiveness of recruitment maneuvers is intimately dependent on lung recruitability. Cressoni and colleagues found that lung recruitability was significantly higher in patients with severe ARDS compared to mild and moderate forms (37). Furthermore, the amount of recruitable tissue between plateau pressures of 30 and 45 cmH_2_O was negligible in mild ARDS, but drastically increased in severe ARDS (8 and 43%, respectively). Recruitability is mainly explained by the distribution of lung lesions, their nature, as well as the timing of onset (8, 38). Firstly, lung morphology (focal or non-focal) rather than the origin of lung disease (pulmonary or extra-pulmonary) may explain the considerable variability in recruitability among patients with ARDS (18, 28, 29, 39). In focal ARDS, recruitment maneuvers might expose the patient to overdistension of already open lung regions, but may be beneficial in non-focal ARDS with more collapsed tissue and a potential higher oxygenation benefit (40).

Several techniques for assessing recruitability have been evaluated and some are still under investigation. Although CT-scan remains the gold-standard in this area, bedside techniques including electrical impedance tomography, lung ultrasound, and recruitment-to-inflation ratio are promising tools (28, 41). A clinically significant difference in efficacy was found when analyzing only correctly classified patients based on lung morphology who received well adapted recruitment maneuvers (29). Secondly, the predominant lesion type could also play a role in the response to recruitment maneuvers (8). Patients with a better oxygenation response to recruitment maneuvers have been shown to have predominantly interstitial edema and compressive and congestive atelectasis (8). Conversely, subjects with minimal oxygenation response had complete alveolar filling with purulent or hemorrhagic material, and consolidation, which are more prominent in direct lung injuries such as pneumonia (8). Thirdly, the timing of the recruitment maneuver relative to the onset of ARDS influences recruitability. Indeed, a change from an exudative to a fibroproliferative process in late ARDS may alter the response to recruitment maneuvers (10, 42). Although clear time cut-offs have not been established, it is unlikely that these maneuvers will be beneficial in patients with ARDS of more than 3 to 5 days who do not have altered chest wall mechanics (10, 42, 43).

## 5. How to perform recruitment maneuvers

Numerous techniques for recruitment maneuvers have been described and are based on the principle of cumulative exposure to higher non-physiological transpulmonary pressures. The optimal recruitment maneuver has not yet been established, but there is evidence to support the use of certain strategies to limit associated complications (9, 31, 43, 44).

Sighs were the first reported recruitment maneuver (45). It consists of the application of a high tidal volume to mimic physiological breathing as it occurs in healthy subjects. The application of three consecutive sighs at a plateau pressure of 45 cmH_2_0 has been associated with improved oxygenation, pulmonary elastance and functional residual capacity in patients with ARDS (46). Yet, the frequency and targeted pressure for these sighs are not standardized in the literature. Despite the reported beneficial effects, efficacy is limited over time and could increase the level of inflammatory markers in the lungs depending on the frequency and volumes applied (45). Benefits of this technique in terms of mortality have not been observed (43).

Sustained inflation is the most studied method and involves the use of continuous positive airway pressure of 30–60 cmH_2_0 for up to 60 s in sedated and paralyzed patients, while monitoring them for possible adverse effects (12, 16, 42, 47, 48). This recruitment method initially showed promise in early clinical trials which demonstrated a survival benefit when used as part of an open lung strategy (12). Sustained inflation has also been shown to be effective in reducing pulmonary atelectasis, improving lung functional variables of oxygenation and mechanics as well as counteracting alveolar derecruitment following airway suctioning (49, 50). However, subsequent studies have brought controversy to its use, highlighting a variable benefit in terms of oxygenation and a marked tendency for patients to become hypotensive, particularly with repeated application (16, 48, 49). In light of the uncertainty surrounding the associated benefits and especially the related hemodynamic complications, the routine use of sustained inflations as recruitment maneuvers is no longer recommended (16, 44). Another argument against the use of sustained inflations as traditionally applied is that time is an important aspect of alveolar recruitment. Indeed, application of 30 cmH_2_O plateau pressure with a PEEP of 5 cmH_2_O for 2 s opens 75% of alveoli, whereas continued recruitment for 40 s increases this proportion to only 85% (13). Consequently, it has been shown that the majority of recruitment occurs within the first 10 s of the recruitment maneuver, whereas hemodynamic impairment becomes significant after 10 s of initiation (51).

To limit the negative hemodynamic consequences and the development of lung injury, some authors have suggested adopting a more conservative approach to alveolar recruitment consisting of a gradual increase in PEEP and/or driving pressure to ensure a more homogeneous pressure distribution throughout the lung parenchyma (9, 43, 44, 52). These approaches include:

- Incremental increase in driving pressure (2-min intervals with 5 cmH_2_0 steps) at a fixed PEEP level (10–15 cmH_2_O).
- Pressure-controlled ventilation applied with a PEEP of 3 cmH_2_O above the upper inflection point of the pressure-volume curve (peak pressure set at 50 cmH_2_O for 2 min), followed by transition to volume-controlled ventilation with a stepwise decrease in PEEP and plateau pressure below the upper inflection point. The final PEEP is set at 3–4 cmH_2_0 above the lower inflection point of the pressure-volume curve.
- Increase of PEEP to 15 cmH_2_O and end-expiratory pauses for a few seconds, twice per minute over a 15-min session.
- The so-called “RAMP” maneuver, which consists of slowly (over 40–50 s) and progressively increasing the inspiratory pressure up to 40 cmH_2_0.

A recruitment maneuver that may have the best clinical impact of all the measures presented above is called the “maximum recruitment strategy”. It consists of 2-min stepwise increases in PEEP of 5–10 cmH_2_O with a constant driving pressure (10–15 cmH_2_0) until a combined value of oxygen and carbon dioxide partial pressures (PaO_2_ + PaCO_2_) above 400 mm Hg is achieved (53). When joined with a decremental PEEP titration, beneficial effects in terms of recruitment of non-ventilated lung regions can be observed up to several days after the recruitment maneuver in patients with early ARDS (54). Interestingly, values of PEEP and peak inspiratory pressures as high as 45 cmH_2_O and 60 cmH_2_0, respectively, can be required with only transient hemodynamic decline and hypercapnia, and without occurrence of significant clinical complications, including barotrauma. Other studies using different recruitment durations as well as maximum peak inspiratory pressure have been investigated with contrasting results, highlighting the lack of consensus on which parameters to use (55, 56).

A shared conclusion in the literature is that following recruitment maneuvers, a PEEP higher than that used before the recruitment maneuver (6–7 cmH_2_0 above baseline) is necessary in order to keep the alveoli open and preserve the beneficial effects on oxygenation over time (8). Besides, manual sustained hyperinflations require a shorter amount of time to perform, whereas stepwise increases in PEEP or tidal volume may prevent untoward subject responses such as straining or coughing when the depth of anesthesia is equivocal (31). Interestingly, in a recent study, sustained inflation followed by application of a PEEP of 5 cmH_2_O compared with stepwise recruitment, were equally effective in improving pulmonary function in dogs undergoing laparoscopic surgery (57).

## 6. Methods to assess the effects of recruitment maneuvers

Closely related to the debate about the efficacy of recruitment maneuvers and their preferred context of use is the question of what criteria to use to identify responders. Some authors have suggested that responders can be identified by an increase in oxygenation and lung compliance and/or a significant reduction in driving pressure after completion of the recruitment maneuver (13). Physiologically, lung recruitment is expected to improve gas exchange and respiratory mechanics as more alveoli participate in tidal ventilation (39). Oxygenation, as measured by PaO_2_ or the PaO_2_/FiO_2_ ratio, is a commonly used indicator to assess the effects of recruitment maneuvers and to distinguish responders from non-responders (18, 39). Setting a PEEP and FiO_2_ to maintain a target oxygen saturation (88–95%) or PaO_2_ (55–80 mm Hg) has been used to identify the best PEEP value, which was associated with decreased mortality (44). However, anatomical recruitment as measured with computed tomography, and oxygenation (termed functional recruitment) have been shown to be poorly correlated with each other (58). Indeed, incremental PEEP during a recruitment maneuver might affect oxygenation by mechanisms other than non-aerated tissue recruitment, such as changes in cardiac output and blood flow distribution in the lungs (58, 59). Improved lung compliance at higher pressures may reflect both improved mechanical properties of already open lung units and recruitment of previously collapsed alveoli (39). The resulting reduction in driving pressure can have a beneficial effect on outcomes. Amato and colleagues suggested that a value below 15 cmH_2_0 for driving pressure may reduce mortality in patients with ARDS (20), which was contradicted in the ART trial where higher mortality was noted despite a decrease in driving pressure from 13.5 cmH_2_O to 11.5 cmH_2_O (21).

A more pragmatic answer to the issue of identifying responders may be to define recruitment as a continuous spectrum that depends on the applied airway pressure and several imaging characteristics (28). Talking about “responders” and “non-responders” might therefore be a false dichotomization, and the terms “high” and “low” responders may be more appropriate (28).

Some limitations should first be noted regarding mechanistic studies of recruitment maneuvers. Confounding factors may bias the interpretation of the efficacy of recruitment maneuvers and include the heterogeneous nature of pulmonary lesions in patients with ARDS, the nature of the injury encountered as well as its severity, the timing of recruitment maneuvers relative to the onset of clinical signs, the strategy of mechanical ventilation prior to initiation of recruitment maneuvers, possible concomitant chest wall lesions, and the prior existence of hemodynamic alterations in the patient (8).

### 6.1. Computed tomography (CT-scan)

CT-scan is the gold standard for assessing pulmonary re-aeration. Recruitment is quantified as the amount of un-aerated tissue at a given pressure that re-inflates at higher pressures (39), and is usually expressed as a percentage of total lung volume (28). With this technique, recruitable areas in patients with moderate to severe ARDS have been estimated at 13 ± 11% of total lung weight with a strong correlation to the severity of lung injury (36). Additionally, a non-focal morphology of ARDS evaluated by CT-scan has been shown predictive of high pulmonary re-aeration after recruitment maneuvers (28). It must be noted that CT-scan only measures the anatomical recruitment of tissues. The main disadvantages associated with it are that it is time consuming, exposes the patient repeatedly to radiation, and cannot be performed at the bedside (39).

### 6.2. Pressure-volume curve

The application of recruitment maneuvers is strongly associated with improved lung compliance (31). Contemporary ventilators provide bedside pressure-volume loops and calculate dynamic compliance of the patient's respiratory system. A verticalization of the pressure-volume curve after implementation of a higher PEEP implies gas recruitment, thus allowing confirmation (or rejection) of the effectiveness of recruitment maneuvers (31, 39). However, compliance might be more related to the improvement or deterioration of already ventilated lung units than the actual recruitment of atelectatic lung units (58). In a human study, a strong correlation was found between the amount of potentially recruitable lung tissue on CT-scan and pressure-volume loop parameters (hysteresis ratio and maximum normalized distance) in patients with early stages of ARDS (59). However, these strategies for assessing pulmonary recruitability have been performed under static conditions (low-flow method) that require deep sedation or paralysis, which is a potential limitation to the use of this technique (59).

### 6.3. Electrical impedance tomography

Electrical impedance tomography is a real-time, radiation-free, non-invasive bedside technique that provides cross-sectional images of the distribution of electrical conductivity within the body (59). A belt equipped with a set number of electrodes is placed around the thorax, which must be at least 60 cm in circumference due to the limited size of the device (60). Briefly, a known alternative current is applied across “driving electrodes” while the electrical impedance tomography system reads the resulting voltage at the other electrodes, which in turn act sequentially as driving electrodes, ultimately generating a reconstructed image based on the impedance changes across thoracic structures (32 × 32 pixel matrix). As most electrical impedance tomography devices create nearly 50 images per second, substantial information on global, regional and pixel-level dynamic ventilatory variables and compliance can be obtained (60). It is then possible to estimate the percentage of recruitable collapsed alveoli by measuring relative changes in pixel compliance (the total impedance change for that pixel divided by the airway pressure) (39). This requires a baseline value at high PEEP and a decremental PEEP trial (39). Decreasing pixel compliance with reduced PEEP indicates alveolar collapse, whereas declining pixel compliance with increasing PEEP indicates local overdistension (39). In practice, predominant ventilation in non-dependent areas could predict greater lung re-aeration after a recruitment maneuver (61). Ultimately, this technique does not provide information about aerated lung tissue like CT-scan, but it does provide data about changes in lung volumes associated with a change in ventilator parameters. Experimental studies in dogs have shown that electrical impedance tomography can identify pulmonary edema, changes in pulmonary air and fluid volumes, and lung hyperinflation (62). A correlation between electrical impedance tomography and CT-scan images has been demonstrated in anesthetized dogs ventilated at different levels of PEEP (62). Clinical and experimental studies in dogs and horses are encouraging for the applicability of electrical impedance tomography in veterinary medicine, particularly to help determine the effectiveness of recruitment maneuvers as well as the optimal level of PEEP to maintain recruitment after said maneuver (60, 63, 64). Still, electrical impedance tomography has several disadvantages. Most importantly, the images created have low spatial resolution compared with CT-scan and magnetic resonance imaging, which limits the ability to provide morphologic information. In addition, while electrical impedance tomography is useful for monitoring lung function over time in a single patient, it may not be suitable for interindividual comparisons. Finally, the need for optimal and stable skin-to-electrode contact over time to avoid artifacts during data collection is a challenge, particularly in thick-coated patients (60, 65). Clinicians should also be aware that pleural effusion and adjacent cardiac structures can cause paradoxical ‘out-of-phase' impedance changes in the surrounding lung tissues due to an overshoot phenomenon introduced by the reconstruction algorithm (66).

### 6.4. Lung ultrasound

Lung ultrasound is another popular, reproducible, and non-invasive imaging modality available at the bedside (28, 67). This semi-quantitative method relies on the assessment of subpleural pulmonary zones, for which an aeration score has been developed in humans based on the presence of B-lines and consolidation (67). Specifically, the observation of anteriorly localized consolidation and crater-like subpleural consolidation predicts a positive response to recruitment maneuvers (i.e., lung recruitability) in patients with ARDS, and a highly significant correlation has been found between PEEP-induced lung recruitment, as measured by pressure-volume curves, and the ultrasound aeration score (28, 67, 68). A four-step algorithm has been proposed in humans using lung ultrasound to guide recruitment maneuvers in practice (69). Firstly, the presence of alveolar collapse is assessed by the identification of simultaneous coalescing B-lines and lung consolidation, as well as a high aeration score (67). If present, a recruitment maneuver is indicated. Secondly, hemodynamic status is assessed by various methods using ultrasound. These can include the caudal vena cava collapsibility index, the transmitral E-wave velocity, the end-diastolic left ventricular internal diameter normalized to body weight or the presence of the papillary muscle kissing sign as demonstrated in dogs (70, 71). Preload dependence, hypovolemia, vasoplegia and impaired myocardial contractility are considered contraindication to the maneuver. Thirdly, detection of the lung opening pressure during the pressure increase of the recruitment maneuver and of the closing pressure during subsequent PEEP titration is performed. The ultrasound probe is positioned in the most dependent region of the atelectatic lung to monitor loss of consolidation pattern. Subsequent evaluation of the contralateral lung is then performed to validate the resolution of consolidation, and the airway pressure at that time is identified as the opening pressure. The reverse logic applies during decremental PEEP titration, allowing identification of the closing pressure, and thus the ideal PEEP. Finally, adjustment of hemodynamic therapies is performed to optimize the improvement of cardiopulmonary function. Ultrasound examination of the lungs may be difficult to perform reliably in obese patients or when subcutaneous emphysema is present (67). In addition, the role of lung ultrasound in assessing alveolar overdistension remains undetermined (28).

### 6.5. Stress index

The stress index analyzes the shape of the dynamic pressure-time curve during volume-controlled ventilation with a constant inspiratory flow (44). A linear increase in pressure corresponds to a stress index equal to 1, suggesting tidal inflation of normally aerated alveoli without overdistension. Tidal recruitment is implied with a stress index <1 (downward concavity of the curve, i.e., compliance increases during tidal inflation), whereas a stress index >1 suggests overdistension (upward concavity of the curve, i.e., compliance decreases during tidal inflation). This method has been mainly used to determine the optimal level of PEEP for a given patient (44). Interestingly, although an overall improvement in compliance and a lower plasma concentration of inflammatory mediators were found using the stress index approach, a resulting alveolar hyperinflation was observed in human patients ventilated with a low tidal volume as per the ARDS network strategy (42). A major limitation of the stress index method is that the lungs and chest wall are coupled in series. Therefore, changes in the extrapulmonary environment can confound its interpretation, especially with ARDS because of the extreme variability in chest wall compliance associated with this condition and the frequent coexistence of pleural effusion (72). In the latter situation, as the chest wall expands outward, the adjacent lung is compressed and may appear falsely overdistended using the stress index method (73).

### 6.6. Recruitment-to-inflation ratio

The recruitment-to-inflation ratio (R/I ratio) is a novel single breath maneuver that can be performed with any mechanical ventilator, developed to assess lung recruitment in patients with ARDS (41). It represents the proportion of volume distributed to the recruited lung to that into the baby lung when PEEP is changed (41). Mathematically, this is equivalent to calculating the ratio between the compliance of the respiratory system at high PEEP when the lung is fully recruited to the compliance of the respiratory system at low PEEP after derecruitment. As a confounding factor for the measurement of alveolar pressure, the airway opening pressure, which corresponds to complete airway closure, should be identified first and, if present, subtracted from the previous low PEEP in the compliance measurement. This method can provide useful information for identifying both the risk of atelectrauma by setting a low PEEP in patients with a high R/I ratio and hyperinflation by setting a high PEEP in patients with a low R/I ratio (41). By defining high recruiters as a ratio greater than or equal to 0.5 and low recruiters as a R/I ratio <0.5, a correlation was found between this ratio and improved oxygenation, alveolar dead space, and hemodynamics. This reference cut-off must, however, be individualized to the different models of ventilators. The R/I ratio provides a promising bedside tool to characterize lung recruitability over a given range of PEEP, which can be used to customize this parameter (41).

## 7. Controversies over recruitment maneuvers

The performance of recruitment maneuvers in patients with ARDS remains highly controversial, particularly in light of the uncertainty about their effectiveness in improving survival (18, 19). From a clinical point of view, and in order to limit the risk of barotrauma, the potential recruitability of the lung tissue must first be determined before implementing these maneuvers. Surprisingly, in most recent randomized clinical trials in humans, no attempt has been made to assess the actual resulting alveolar recruitment from these maneuvers (21, 22, 55). As discussed above, an undeniable reason for this is that bedside assessment of lung recruitment is difficult, and the applicability of recently developed methods (lung ultrasound, electrical impedance tomography, R/I ratio) remains to be defined (74, 75).

In people, the positive effects of lung recruitment maneuvers on oxygenation (quantified by the PaO_2_/FiO_2_ ratio or PaO_2_ level) and lung compliance are well established in patients with and without ARDS (31). Nevertheless, recent data lack strong evidence for their routine usage because of the adverse effects associated with their use (17–19, 25, 26). Positive effects arise from the homogenization of pulmonary ventilation by the recruitment of new lung units, which leads to an increase in lung compliance, easier work of breathing, and a decrease in the driving pressure needed to inflate alveoli. Thereby, gas exchange is optimized. Negative effects are closely related to the increase in intrathoracic pressure. Consequences include VILI (*via* the above-mentioned mechanisms), overdistension of aerated regions causing a loss in lung compliance, and hemodynamic impairment (18).

Hemodynamic tolerance is a key element in the decision to perform a recruitment maneuver (18, 27). Increased intrathoracic pressure during recruitment maneuvers transiently compromises hemodynamic function by decreasing right and left ventricular preload and increasing pulmonary vascular resistance and right ventricular afterload, resulting in decreased cardiac output and arterial blood pressure, and increased heart rate (76). In patients with low vascular filling, an increased risk of post-maneuver cardiac arrest was reported in the ART trial, again emphasizing the importance of limiting pressure levels applied to the lungs (18, 21). In patients with ARDS, recruitment maneuvers were also associated with the development of cardiac arrhythmias in a recent trial (22). Consequently, prior hemodynamic stabilization and close monitoring should be sought before performing a recruitment maneuver to improve the tolerability of the procedure given the cardiopulmonary interdependence (27).

Interestingly, several meta-analyses in patients with moderate to severe ARDS demonstrate that recruitment maneuvers do not significantly increase the rates of pneumothorax, pneumomediastinum, or subcutaneous emphysema secondary to alveolar rupture (18, 19, 25, 26). It should be noted, however, that quantification of overdistension is highly dependent on the method used. Up to 20% of the lungs may be overdistended when assessed by CT-scan (28). Using electrical impedance tomography, Karsten and colleagues demonstrated the presence of overdistension in 5–30% of patients with ARDS (77). Local overdistension in non-dependent lungs could even exceed 60% in some areas (78). Thus, even if recruitment maneuvers do not ultimately lead to alveolar rupture, inflammatory changes undetectable by bedside techniques (biotrauma) may develop and worsen the patient's respiratory function (79). The ART trial found an increased rate of barotrauma with recruitment maneuvers, but their use of high airway pressures, up to 60 cmH_2_O during the sustained inflation maneuver, may be the primary cause of this outcome (21, 22). In a recent meta-analysis, lung recruitment maneuvers reduced the incidence of postoperative pulmonary complications in patients under general anesthesia, but these results have limited implication due to high heterogeneity (24). Interestingly, this study found that a more sustained recruitment maneuver was associated with a decrease in postoperative pulmonary complications compared with a stepwise approach (24).

The use of recruitment maneuvers does not appear to significantly improve the duration of mechanical ventilation, hospital or ICU length of stay in people (18, 25, 26). However, it has been associated with less frequent use of rescue therapies in most recent randomized controlled trials (18).

A major point of debate on recruitment maneuvers is their effect on survival in patients with ARDS. Interestingly, when considering randomized controlled trials in people performed between 2008 and 2017, a significant reduction in 28-day mortality was found in association with the use of recruitment maneuvers (18). However, the conclusions of these studies must be interpreted in context of their nuances. First, a meta-analysis of these studies showed an improvement in 28-day survival, but did not reach the required information size to conclude this with a high level of certainty (19). Similarly, a systematic review that reported a decrease in ICU mortality but not at any at other time points on follow-up, graded the quality of evidence as low because of various co-interventions that might have interfered with clinical outcome (high PEEP strategy, PEEP titration) (80). The beneficial effect on mortality was no longer observed in a meta-analysis that included the most recent studies published after 2017, in particular the ART (21), PHARLAP (22) and LIVE (29) trials (17, 18, 25, 26). This latter fact is further supported by the information that two of the recent meta-analyses included in the assessment performed a trial sequential analysis to measure the risk of random errors due to data sparsity and multiple testing, strengthening their conclusions (18, 26).

The difficulty of establishing strong recommendations on the use of recruitment maneuvers is emphasized by the heterogeneity of techniques used between randomized controlled trials and therefore of maximum airway pressures applied (from 35 to 60 cmH_2_O), and the existence of co-interventions (PEEP, prone positioning, neuromuscular blockade) with associated consequences on oxygenation, ventilation efficiency and adverse effects (18). Lung recruitability has also been poorly interrogated in the majority of randomized controlled trials, which may lead to variable responses depending on lung morphology in ARDS (74). On this topic, a recent study was designed to test whether a personalized mechanical ventilation strategy based on individual lung morphology would improve survival in patients with ARDS compared with a conventional lung protective strategy (29). This study used CT-scan and patients with focal disease were assigned a low PEEP and high tidal volume, while patients with non-focal disease received a higher PEEP and recruitment maneuvers. Despite disappointing crude results showing no improvement in mortality, misclassification in nearly 21% of cases and high mortality among these patients underscore the difficulty of prospective phenotyping of lung morphology in patients with ARDS. Interestingly, the per-protocol analysis of patients with correctly classified lung morphology at inclusion showed a significant increase in survival for those in the personalized group (29).

## 8. Recommendations for the use of recruitment maneuvers

Considering the many physiological and clinical downsides of recruitment maneuvers, and the lack of evidence regarding clinical outcomes despite 25 years of studies, it is best to consider an individualized rather than systematic use of recruitment maneuvers in patients with ARDS; one strategy does not fit all (18, 27). Alveolar recruitment is desirable first if it can be performed safely, which requires prior assessment of lung recruitability (8). As noted above, the precise context in which to consider performing recruitment maneuvers or even how to proceed is not yet clearly defined. The balance between positive effects of recruitment maneuvers (improvement of oxygenation and lung compliance) and negative consequences (overdistension and hemodynamic risks) must always be emphasized in decision making.

As a general rule, and only in the setting of severe ARDS, performing recruitment maneuvers as rescue therapy should be reserved for a minority of patients with refractory hypoxemia demonstrating good lung recruitability. There also appears to be specific situations in which recruitment maneuvers may be appropriate, such as in morbidly obese patients or cases of intraabdominal hypertension (8).

Regarding the recommended way to perform recruitment maneuvers, a stepwise approach might be preferred to sustained inflation, mainly because of the adverse hemodynamic consequences associated with the latter (44). This allows for individualization of the pressure applied to each patient and termination of the maneuver in case of hypotension, desaturation, or barotrauma (19, 44). There is no consensus on what plateau pressure to achieve during a recruitment maneuver. Even at a value of 45 cmH_2_0, ~25% of the lungs remains collapsed in moderate to severe ARDS, but higher values may increase the risk of volutrauma and barotrauma (37). Therefore, reaching threshold opening pressure in ARDS does not equate to complete recruitment, and a proportion of atelectrauma may be accepted (8, 37). If the recruitment maneuver is effective, sufficient PEEP is required to maintain recruitment (8, 44). However, finding the optimal PEEP after a recruitment maneuver is challenging and matter of debate, as it may depend on the regional distribution of lung lesions, characteristics of the atelectasis (congestive or consolidation), as well as the method used to recruit the lungs (25). To reduce derecruitment in the acute phase of ARDS, a minimum PEEP of 10–12 cmH_2_0 should be implemented with conventional mechanical ventilation strategies, with values >20 cmH_2_0 necessary in severe cases, including patients with a chest wall compliance defect (8). Determination of the ideal PEEP is beyond the scope of this review and will be developed elsewhere.

Recruitment maneuvers are contraindicated in hemodynamically unstable patients (especially with right-sided heart failure), those with intracranial hypertension, pneumothorax or a predisposition to barotrauma, and more generally in patients with focal lung pathology (8, 81).

During general anesthesia, depending on the type of surgery and the patient's condition, the clinician may use alveolar recruitment maneuvers followed by PEEP after induction of general anesthesia with endotracheal intubation (31). Interestingly, one study demonstrated the superiority of sustained inflation over the stepwise recruitment maneuver in reducing postoperative complications in the general population undergoing general anesthesia for various indications (24). Current data do not support the hypothesis that the benefit of alveolar recruitment maneuvers extends significantly into the postoperative period (31). Several situations in the context of general anesthesia and/or mechanical ventilation, such as endotracheal suctioning and ventilator disconnection, which can lead to alveolar derecruitment and atelectasis formation, may benefit from recruitment maneuvers (82).

## 9. Adjunct therapies and alternative ventilatory modes

As stated above, mortality from refractory hypoxemia remains unacceptably high in patients with severe ARDS (1). Even with an open-lung ventilation strategy, a select minority of patients still experience profound hypoxemia. Therefore, clinicians may consider a number of rescue therapies as temporary modalities to support or replace the respiratory system, while accepting higher risks and lesser evidence for their use compared with standard lung protective ventilation (1). Rescue therapies include prone positioning, use of neuromuscular blockade, inhaled pulmonary vasodilators, extracorporeal membrane oxygenation, and unconventional ventilatory modes (1, 5, 38).

### 9.1. Prone positioning

Delivery of mechanical ventilation with patient in prone position is defined as prone ventilation (83). In the supine position, the weight of the ventral lungs, mediastinal structures and abdominal viscera increases the pleural pressure in the dorsal lungs, promoting alveolar collapse (13). Prone positioning changes the gravitational forces which promotes re-aeration of the now non-dependent dorsal lungs. Moreover, regional diaphragmatic movements homogenize global pulmonary ventilation, improve ventilation-perfusion matching *via* anterior displacement of mediastinal structures, reduce the ventral-dorsal transpulmonary pressure difference, enhance mobilization of secretions, and thus reduce the likelihood of VILI compared with supine positioning (1, 5, 38, 83, 84).

The newly opened dorsal lung regions, despite their non-dependent orientation, remain well perfused, thereby decreasing intrapulmonary shunting (5). Evidence in people also suggests that proinflammatory cytokine release may be reduced following the procedure (85). Prone positioning requires no specific equipment, but should be performed by a well-trained team (5). The landmark PROSEVA trial demonstrated a significant increase in survival in patients with severe (PaO_2_/FiO_2_ ratio ≤150 mm Hg, FiO_2_ = 0.6, PEEP ≥ 5 mm Hg), early (≤36 h) ARDS, ventilated according to a lung protective protocol combined with neuromuscular blockade. Some authors have thus suggested minimum 12 to 16-h daily prone sessions with the head of the bed elevated 30–45° to limit head edema and gastroesophageal reflux (1, 15, 86).

Other important aspects proposed for successful implementation of prone positioning include appropriate prior titration of PEEP, careful use of neuromuscular blocking agents and sedative drugs to avoid diaphragmatic paralysis, and discontinuation of prone positioning when sustained improvement of oxygenation is observed (86). Most complications related to prone positioning in people arise when the patient's position is changed, including accidental removal of the endotracheal tube, drains, or catheters (5, 38). Prone positioning should not be implemented in patients in shock, or with unmonitored intracranial hypertension, severe traumatic injuries or spinal instability (84). In dogs with experimentally induced ARDS, a protective ventilation strategy combined with recruitment maneuvers was safer in the prone position than in the supine position (87). However, no studies to date have examined the value of altering the positioning of cats and dogs during mechanical ventilation in the clinical setting of refractory hypoxemia.

To overcome the technical issues associated with prone positioning or when contraindicated, it has recently been proposed that sequential lateral positioning of people with ARDS can be used as a recruitment maneuver for each lung, with subsequent application of sufficient PEEP to prevent derecruitment (88). This method of shifting the patient from the supine to both lateral positions, improved respiratory mechanics and gas exchange as evaluated by electrical impedance tomography measurements and lung ultrasound-based consolidation scores (88). The resulting lung re-expansion was maintained for at least 30 min after return to the supine position. This procedure was not associated with increased airway pressure and did not demonstrate hemodynamic side effects, but its long-term superiority to prone positioning remains unknown. It should therefore not yet be considered as an equivalent alternative. Cats and dogs are naturally in the prone position during mechanical ventilation. In dogs with experimentally induced ARDS and VILI, prone positioning resulted in a less severe and more homogeneous distribution of VILI and reduced histologic changes compared with supine positioning (89, 90). Based on the aforementioned underlying physiology, sequential lateral positioning might also be beneficial in veterinary practice and requires further investigation.

### 9.2. Neuromuscular blockade

Neuromuscular blocking agents are used in hypoxemic patients with poor ventilator synchrony despite deep sedation (86). Physiologically, improved synchrony may result in more uniform lung recruitment and improved compliance and gas exchange (5, 38). The positive effects of neuromuscular blocking agents could also be related to a decrease in biotrauma (91). In patients with ARDS randomized to receive cisatracurium, proinflammatory cytokine levels in serum and bronchoalveolar lavage fluid were significantly lower than in the control group (91). Similarly, treatment with a 48-hour continuous infusion of cisatracurium in patients with severe early ARDS (PaO_2_/FIO_2_ ratio <150 mm Hg) significantly reduced mortality and barotrauma, while increasing the number of ventilator-free days (92). However, recent data from a large-scale randomized-controlled trial, conducted with the same protocol as the aforementioned ACURASYS trial, have challenged its conclusions by demonstrating a lack of benefit in terms of 90-day survival (93).

These potential benefits should be weighed against neuromuscular blockade-related progressive atelectasis due to loss of diaphragmatic tone with resulting hypoxemia and, more importantly, intensive care unit-acquired weakness (1, 38). Critical illness polyneuropathy and post-traumatic stress disorders have been associated with the use of neuromuscular blocking agents in people, suggesting limiting their use to the shortest possible duration and providing appropriate sedation to avoid inadvertent awake paralysis in ventilated patients (1). Moreover, because most randomized controlled trials have been conducted using cisatracurium, the recommendation for the use of a neuromuscular blocking agent during ARDS is limited to this agent (86). Recently, the safety profile of vecuronium has been shown to be comparable to cisatracurium in people with ARDS, with no significant difference in efficacy (94). In cats and dogs with ARDS, the optimal strategy for determining proper sedation before neuromuscular blockade remains unexplored (95).

### 9.3. Inhaled pulmonary vasodilators

During ARDS, the use of inhaled pulmonary vasodilators has two purposes. First, they aim to reverse the pulmonary hypoxic vasoconstriction that occurs naturally in healthy alveoli. Second, because increased pulmonary vascular resistance due to pulmonary vasoconstriction and atelectasis can lead to right-sided heart failure, inhaled pulmonary vasodilators indirectly support the right ventricular function (96). Inhaled pulmonary vasodilators theoretically act in well-ventilated lung units, helping to redirect blood flow away from poorly ventilated lung areas and improve V/Q mismatch (38). Advantages of the inhaled route include its selective delivery to well-ventilated lungs and ease of administration (5, 38). Although temporary benefits toward oxygenation have been documented with their use, they were not attributable to improved lung function, reduced lung injury, or resolution of the underlying cause of ARDS (38). Studies performed during ARDS have not demonstrated any benefit in terms of mortality (96).

Two classes of molecules have been used, inhaled nitric oxide and prostaglandins (mainly epoprostenol), both with short half-lives (96). The use of nitric oxide has been associated with several adverse effects, including methemoglobinemia, kidney failure, inhibition of platelet activity and hypotension. It also requires a specialized delivery system (96). As a result, its use has been largely replaced by that of prostaglandins, which are less expensive and easily administered *via* a nebulizer connected to the mechanical ventilation circuit (38, 96). The adverse effects of prostaglandins are also fewer than those of nitric oxide (38). Besides improving oxygenation, they reduce pulmonary vascular pressure and may be useful in patients with preexisting pulmonary hypertension (96). The use of inhaled pulmonary vasodilators should be done with the understanding that severe rebound hypertension may happen if the medication is stopped too quickly (96). For epoprostenol, halving the dose every 2–4 h is considered a safe approach in humans (96). Routine use of inhaled pulmonary vasodilators is currently not recommended in people based on existing evidence, but may be considered as a temporary measure before a more definitive intervention such as extracorporeal membrane oxygenation in patients with severe ARDS and refractory hypoxemia (1, 38, 96).

### 9.4. Extracorporeal membrane oxygenation

Extracorporeal membrane oxygenation is a technology used in humans in which blood is drained via the superior or inferior vena cava and reinfused into the right atrium after circulating through an artificial lung that ensures oxygenation and carbon dioxide removal by the process of diffusion (1, 38). In individuals with profound hypoxemia (PaO_2_/FiO_2_ ratio <80 mm Hg) or severe uncompensated hypercapnia with acidemia (pH <7.15) resistant to conventional low-volume, low-pressure ventilation, prone positioning and inhaled pulmonary vasodilators, veno-venous extracorporeal membrane oxygenation allows for low-tidal volume protective ventilation combined with a lower FiO_2_, thereby limiting the main injury mechanisms associated with VILI (i.e., barotrauma, volutrauma, biotrauma, oxygen toxicity and atelectrauma) (38, 86). Tidal volume is also significantly reduced (almost halved) leading to a substantial reduction in plateau pressure without worsening derecruitment as PEEP is kept constant (5, 38). However, extracorporeal membrane oxygenation management requires an experienced team, especially with regard to complications related to the intense anticoagulation required to avoid clotting in the circuit, and the associated risks of bleeding (86). It is generally used as a last resort in people with severe ARDS, and should not be implemented in patients ventilated for more than 7 days, or patients with multiple organ failure, that are not candidates for lung transplant, or have absolute contraindications to anticoagulation (86).

Data are still controversial regarding clinical benefit, although a trend toward decreased mortality and renal failure was observed in the CESAR trial, which compared early use of extracorporeal membrane oxygenation with conventional ventilation strategies in patients with severe but reversible respiratory failure (97). Unfortunately, the lack of standardized ventilation protocols limits the strength of the evidence. More recently, the EOLIA trial failed to demonstrate a mortality benefit in the extracorporeal membrane oxygenation group compared with the conventional therapy group (98). Further studies are needed to make a definitive recommendation for or against the use of extracorporeal membrane oxygenation in patients with severe ARDS (5).

While these rescue therapies may be considered to improve the effectiveness of mechanical ventilation and reduce the risk of VILI, unconventional modes of ventilation may also be considered.

### 9.5. Airway pressure release ventilation

Airway pressure release ventilation (APRV) is a time-cycled, pressure-controlled, inverse ratio ventilatory mode based on the concept of open lung ventilation, which relies on the application of high continuous positive airway pressure to promote and maintain alveolar recruitment, with a short phase of intermittent release to a lower pressure allowing ventilation (99, 100). Importantly, it also allows unrestricted spontaneous breathing throughout respiration (100). This mode is available on most modern ventilators (100). Theoretical benefits of the APRV mode include : (1) Lung protective recruitment by decreasing the frequency of repetitive inflation/deflation of the lungs, improving ventilation in non-dependent areas through longer inspiratory duration, creating a stabilized open lung using lower pressure compared with conventional modes, and limiting atelectrauma through partial and short emptying of the lungs during the release phase. (2) Improved patient-ventilator synchrony. (3) Improved V/Q mismatch, decreased pulmonary vascular resistance, improved respiratory compliance, cardiac index and oxygen delivery due to unrestricted spontaneous breathing. (4) Reduced need for sedation and neuromuscular blockade, thus theoretically leading to lower intensive care unit-related delirium or neuromuscular blocking agent-related myopathy. (5) Protection against ventilator-associated pneumonia, which has been primarily observed in humans with trauma suffering from pulmonary contusions (91). This was postulated to occur due to improved lung recruitment, less sedative use, and better mucus clearance through higher expiratory than inspiratory flow rates (100). In practice, there are four basic settings to control in APRV other than FiO_2_ (101–103) :

- High-level pressure (P_High_): Analogous to continuous positive airway pressure, inspiratory pressure similar to plateau pressure, typically set initially as the patient's plateau pressure on a conventional mode prior to initiation of APRV. Common values oscillate between 25 and 35 cmH_2_O.
- High-pressure time (T_High_): Duration of inspiratory time; combined with P_High_, is referred to as the CPAP phase, which influences oxygenation. Typically ranges from 3 to 8 s, but can be set as low as 1.5 s in severe cases of ARDS; set at 4 s initially. It is commonly set to occupy 90% of the total cycle time.
- Low-level pressure (P_Low_): Expiratory pressure similar to PEEP, typically set at 0 cmH_2_O to achieve the greatest pressure differential between P_High_ and P_Low_.
- Low-pressure time (T_Low_): Duration of expiratory time, prevents derecruitment; combined with P_Low_, is referred to as the release phase, which influences carbon dioxide removal. Typical values range from 0.2-0.8 seconds; set at 0.5 seconds initially.

While P_High_ over a prolonged period allows slow alveolar recruitment, T_Low_ prevents alveolar collapse. The short release period terminates the expiratory flow early, permitting only partial unloading of lung capacity, thus causing auto-PEEP and preventing alveolar instability (101). There are two main strategies when setting APRV, the fixed setting technique and the personal setting approach (100). The latter is also known as time-controlled adaptative ventilation (TCAV^TM^) and is the most widely used (100). In this method, the time spent at plateau pressure covers about 90% of the respiratory cycle. T_Low_ is set so that the end-expiratory flow/peak-expiratory flow ratio equals ~75%, thus preventing alveolar collapse. Accordingly, time rather than pressure controls the end-expiratory lung volume. Continuous analysis of the expiratory flow curve is therefore necessary as respiratory mechanics change during ventilation, in order to accurately and independently set and adjust T_High_ and T_Low_ (100, 104). If hypoxemia is present, an increase of P_High_ then of T_High_ are warranted. Only as a last resort, FiO_2_ should be increased. Hypercapnia can be tolerated if pH remains above 7.25 and there are no adverse effects of acidosis (i.e., permissive hypercapnia) (103). Otherwise, a decrease of T_High_ is indicated while ensuring that the respiratory circuit is free of secretions or excessive moisture. An increase in P_High_ can also be considered in order to maximize recruitment and minimize dead space. If hypocapnia is present, and with adequate cardiac output, an increase in T_High_ should be done (103). To wean a patient off APRV, the FiO_2_ must first be reduced, before gradually decreasing P_High_ while simultaneously increasing T_High_ progressively at each step once P_High_ reaches 20 cmH_2_O. The patient can then be either weaned to a continuous positive airway pressure mode or switched to a conventional pressure-assisted mode, and weaned conventionally thereafter (103).

The survival benefits of APRV use during ARDS are still unproven and subject to speculation. Most randomized controlled trials comparing APRV to conventional, low-tidal volume, lung protective ventilation have small sample sizes and have evaluated heterogenous patient populations, limiting the strength of their conclusions (5, 101). In summary, the main benefits seen with APRV in patients with severe ARDS are mostly short-term endpoints, such as an improvement in oxygenation, respiratory mechanics, possible decrease in hospital length of stay and ventilation requirements. Yet, no randomized controlled trial has proven with a good level of evidence a significant survival benefit, both in adults and in pediatrics (5, 101). However, in a recent meta-analysis, APRV was associated with lower in-hospital mortality with no significant effect on hemodynamics or risk of barotrauma, but heterogeneity was high amongst patients and methodology between trials (105). Therefore, in the absence of a large, multicenter, randomized controlled trial demonstrating a patient outcome benefit compared to low-tidal volume ventilation in patients with ARDS, a definitive recommendation for the use of APRV cannot be made (5, 101, 102). There have been many criticisms of APRV, regarding the difficulty of mastering this mode, the assumed risk of barotrauma, increased right ventricular afterload, or uncontrollable auto-PEEP and dynamic hyperinflation (104). However, a recent review highlighted the fact that some authors' beliefs about APRV are often based on little clinical experience with its use, and are generally not supported by rigorous studies. Although APRV has not yet shown to be superior to low-tidal volume ventilation in multicenter randomized controlled trials, the reverse is equally valid (104).

Information on the use of APRV in animals has been obtained primarily in experimental settings (101). However, two case reports in veterinary medicine suggest that this strategy is applicable for the management of hypoxemia due to aspiration pneumonia and refractory hypercapnia secondary to noncardiogenic pulmonary edema (106, 107). While the first patient was switched to APRV after several days of conventional mechanical ventilation, the second patient was settled after a few hours of initiation of mechanical ventilation. In the early case report, transition from pressure-controlled ventilation to APRV was accomplished by gradually increasing the inspiratory:expiratory ratio over 24 h, although there is no evidence in the human literature that such a delay is necessary (106). Both patients recovered fully. Although more substantial data are needed in veterinary medicine to determine the efficacy of APRV with ARDS, these studies validate its applicability in dogs.

### 9.6. High frequency oscillatory ventilation

High frequency oscillatory ventilation delivers a low amplitude and high frequency tidal volume in combination with maintaining a high end-expiratory pulmonary pressure to decrease alveolar collapse (1, 5, 99). This mode has a strong theoretical basis, as it could achieve most goals pursued by lung protective ventilation strategies, including a more homogeneous distribution of ventilation by maintaining a high mean airway pressure, while reducing the risk of VILI and hyperinflation (1). However, two randomized controlled trials and a recent meta-analysis in humans failed to show any mortality benefit in adults with moderate to severe ARDS as compared with a lung protective strategy. In addition, increased need for sedatives, paralytics, and vasopressors was associated with its use (108–110). Safety profiles have even shown that high frequency oscillatory ventilation was associated with a trend toward increased risk of barotrauma and unfavorable hemodynamics (107). Therefore, guidelines in humans state that high frequency oscillatory ventilation should not be a routine practice in adults or pediatrics with hypoxemic respiratory failure (15, 108, 111).

## 10. Conclusion

In this review, the rationale for and against the use of recruitment maneuvers in light of the recent literature was summarized. Although recruitment maneuvers have a role to play in patients with severe ARDS with diffuse morphology and refractory hypoxemia, their physiological and clinical drawbacks and the heterogeneous nature of respiratory failure call for an individualized rather than routine use. Recruitment maneuvers in ARDS have been primarily associated with improved respiratory mechanics and oxygenation at the expense of hemodynamic stability, but the beneficial effects on mortality are not well established, perhaps due to the lack of a reliable tool to select appropriate candidates. Further research should aim to clarify the exact definition of responders and non-responders, given the increasingly available non-invasive imaging techniques at the bedside to assess both recruitability and response to alveolar recruitment. There is growing evidence that stepwise recruitment maneuvers may be more appropriate in patients with ARDS than sustained inflations, but these observations have not been established in routine general anesthesia. In humans, evidence-based management of ARDS supports the use of lung protective ventilation and prone positioning. Outside of proning sessions, the specific circumstances in which the use of the aforementioned rescue therapies (i.e., neuromuscular blocking agents, inhaled pulmonary vasodilators and extracorporeal membrane oxygenation) is beneficial remains to be determined. As for veterinary medicine, available data on recruitment maneuvers and rescue therapies are mostly limited to experimental studies or individual case reports. APRV is a promising unconventional ventilation strategy, suitable for both dogs and people, whose main advantage is that it allows spontaneous ventilation. The application of its use in cats remains to be determined.

## Author contributions

FB and VG contributed to conception and design of this review. VG searched the literature and organized the database. FB wrote the first draft of the manuscript. All authors contributed to manuscript revision, read, and approved the submitted version.
